# The Gut–Brain Axis as a Therapeutic Target in Multiple Sclerosis

**DOI:** 10.3390/cells12141872

**Published:** 2023-07-17

**Authors:** Ana Maria Buga, Vlad Padureanu, Anca-Lelia Riza, Carmen Nicoleta Oancea, Carmen Valeria Albu, Alexandru Dan Nica

**Affiliations:** 1 Department of Biochemistry, University of Medicine and Pharmacy of Craiova, 200349 Craiova, Romania; ana.buga@umfcv.ro (A.M.B.); carmen.oancea@umfcv.ro (C.N.O.); 2 Department of Internal Medicine, University of Medicine and Pharmacy of Craiova, 200638 Craiova, Romania; vlad.padureanu@umfcv.ro; 3 Laboratory of Human Genomics, University of Medicine and Pharmacy of Craiova, 200638 Craiova, Romania; anca.costache@umfcv.ro; 4 Regional Center for Medical Genetics Dolj, Emergency County Hospital Craiova, 200638 Craiova, Romania; 5 Department of Neurology, University of Medicine and Pharmacy of Craiova, 200349 Craiova, Romania

**Keywords:** gut–brain axis, oxidative distress, neuroinflammation, inflammasomes

## Abstract

The CNS is very susceptible to oxidative stress; the gut microbiota plays an important role as a trigger of oxidative damage that promotes mitochondrial dysfunction, neuroinflammation, and neurodegeneration. In the current review, we discuss recent findings on oxidative-stress-related inflammation mediated by the gut–brain axis in multiple sclerosis (MS). Growing evidence suggests targeting gut microbiota can be a promising strategy for MS management. Intricate interaction between multiple factors leads to increased intra- and inter-individual heterogeneity, frequently painting a different picture in vivo from that obtained under controlled conditions. Following an evidence-based approach, all proposed interventions should be validated in clinical trials with cohorts large enough to reach significance. Our review summarizes existing clinical trials focused on identifying suitable interventions, the suitable combinations, and appropriate timings to target microbiota-related oxidative stress. Most studies assessed relapsing–remitting MS (RRMS); only a few studies with very limited cohorts were carried out in other MS stages (e.g., secondary progressive MS–SPMS). Future trials must consider an extended time frame, perhaps starting with the perinatal period and lasting until the young adult period, aiming to capture as many complex intersystem interactions as possible.

## 1. Introduction

Multiple sclerosis (MS) is a common autoimmune disease that affects the brain and the spinal cord of young adults. According to the Multiple Sclerosis International Federation, MS affects over 1 million active people in Europe, and the number has increased since 2017 [1]. Currently, MS remains a destructive disease without a clear etiology, with high individual, healthcare, and social costs. Since MS incidence is higher in women, it seems to be caused by hormonal factors alongside genetic and environmental factors [2,3]. A demographic study showed that MS incidence is higher in geographic areas further away from the equator (e.g., Western Europe, New Zealand/Australia, or North America) [4]. Recently, a study reported significant changes in MS prevalence related to race and ethnicity [5].

The interaction between determining factors remains elusive. Recent development in understanding the role of gut dysbiosis in the ethio-pathogeny of MS opens a new perspective on the gut–brain link [6,7]. In this light, the gut microbiome is hypothesized to play a central role in mediating these interactions, potentially being involved in the MS risk profile and/or modulating disease progression.

The host microbiome plays a crucial role in immune response, defense against pathogens, and proper body homeostasis. Abnormal interactions between the microbiome and the host are associated with increased systemic inflammation and an altered immune response [8,9]. Dysregulation of the gut–brain axis appears in autoimmune disorders and is related to immune and redox homeostasis imbalances [10,11]. The brain is highly susceptible to oxidative stress that leads to chronic inflammation, which finally leads to demyelination and neurodegeneration [12,13,14,15]. Many studies have disclosed that inflammasome-mediated neuroinflammation is active in neurodegenerative disorders. Aberrant activation of inflammasomes contributes to an uncontrolled inflammatory response. This mechanism promotes the progression of autoimmune diseases. The role of inflammasome activation in relation to MS is still poorly understood. Many cellular and molecular events trigger inflammasome activation mediated by oxidative stress [16,17,18,19]. Reactive oxygen species (ROS) production, mitochondrial disfunction, cellular damage, or inflammation can trigger inflammasome activation.

Recent studies have investigated the role of gut microbiota, the gut–brain axis, and oxidative stress in neurodegenerative and neuropsychiatric disorders [11,18,19,20]. The aim of this review is to explore more deeply the role of the gut–brain axis in oxidative-stress-mediated inflammasome activation in MS. Furthermore, we review recent progress regarding gut–brain axis modulatory potential interventions that directly target host–microbiome interaction, and which also indirectly decrease inflammasome activation by controlling oxidative stress in MS. In the current review, we address several pivotal questions regarding the gut–brain axis as a contributor to MS, which are focused on three main directions. First, increasing evidence shows that gut dysbiosis alters redox signaling and accelerates neurological disease onset through the gut–brain axis. Therefore, we discuss the potential role of the gut–brain axis in demyelination and neurodegeneration in MS, with a focus on gut dysbiosis and gut microbiota active metabolites. Second, we review the recent findings about the role of the gut–brain axis in the brain’s vulnerability to oxidative-stress-related inflammation, with a focus on host inflammasome activation in MS. Third, epidemiological data show that environmental factors significantly impact autoimmune diseases. In this light, we summarize the recent insights linking external factors with gut-related metabolites and MS.

### 1.1. The Brain or the Gut—Which Comes First in MS?

In MS, the host immune system attacks the spinal cord and brain tissue, causing irreversible damage. Trained/innate immunity and the gut make significant contributions to MS. Many gene mutations are related to MS, but not all people with these mutations develop MS. Additionally, there is an overlapping role of the genetic background, the exact time and duration of the triggers’ action, and/or environmental factors. Recent mounting evidence suggests that immune system activation in MS originates from the gut. Other studies report that altering the immune response in MS triggers the gut microbiota changes [21]. Establishing the trigger event is elusive: is it the inflammation of the intestine or that of the brain?

Possible hypotheses are:

(1) Gut dysbiosis leads to CNS inflammation via damage to the intestinal barrier (leaky gut) and activation of the immune system.

(2) Systemic inflammation alters communication between the CNS and enteric nervous system (ENS) via the vagus nerve route, affecting the gut [22]. It is well-known that the vagus nerve controls cytokine production and has immunomodulatory properties [23]. The systemic pro-inflammatory cytokine production is increased and the response of the vagus nerve is decreased. This mechanism leads to gut inflammation and alters intestinal homeostasis [24]. This communication is bidirectional. First, the vagus nerve can detect the microbiota metabolites and transmit this information to the brain, and that can trigger an adaptative or innate immune response [25]. These immune responses aim to modulate microbiota composition [25].

The ability of the gut to maintain its boundaries is an important factor aimed to prevent “bad players” from passing through. It has been recently speculated that the first important event to occur in neurodegenerative diseases is damage to the intestinal gut barrier (GIB) [22]. Leaky gut is associated with systemic inflammation that leads to CNS inflammation. Additionally, growing evidence indicates that the gut lymphoid tissue is one of the main sites of T cell activation [26]. The activation process seems to be closely related to the microbiota and occurs seemingly far from the brain. In this scenario, alteration of the intestinal microbiota or a particular pathogen can trigger an immune response in the gut. A recent study showed that microbiome transplantation in MS patients promotes abnormal T cell activation, which led to encephalitis in mice [27]. This hypothesis relies on proof of the active communication between the CNS and the ENS through the gut–brain axis [26,27].

### 1.2. Is the Vagus Nerve the Route of Microglia Activation in MS?

From a structural and functional point of view, the ENS resembles the CNS. It is made up of nerve fibers (such as the vagus nerve), components of the immune system, as well as signaling molecules (e.g., neurotransmitters, bacterial metabolites), and controls the local immune function. The vagus nerve is responsible for signal transmission to the CNS through afferent fibers, and back through efferent fibers, and has a direct influence on mood and behavior [28].

The microbiota from the gut communicates with the brain through chemical messengers or cells that pass the blood–brain barrier (BBB) [29]. Recent studies on mice report that microbiota signals affect the innate immune response in the CNS and are critical for microglial homeostasis [26,30]. The vagus nerve is one of the key linkers and regulators between the immune system and the CNS. The vagus nerve and components of systemic inflammation serve as routes to extend inflammation beyond the gut, to the CNS [31]. Recent evidence from a cuprizone animal model shows that vagotomy leads to an overactivation of inflammatory response that can be responsible for demyelination [28]. In contrast, stimulation of the vagus nerve decreases cell injuries by decreasing cytokine production and decreases tissue inflammation by regulating the cholinergic immune system in the CNS [23,32,33]. This action is mediated by the α7 nicotinic acetylcholine receptor of the mitochondria in the brain [32,33].

### 1.3. Is the Gut Environment Responsible for CNS Environmental Changes in MS Pathogenesis?

In vivo animal studies have provided the first evidence regarding the direct impact of the intestinal microbiota on the CNS. Undoubtedly, there are significant differences between human MS and laboratory-induced disease. One important difference is the genetic background of inbred animals. However, despite differences, these models are useful to test disease concepts or intervention strategies [34].

There are three available MS animal models: experimental autoimmune encephalomyelitis (EAE), virally induced chronic demyelinating disease (TMEV), and toxin-induced demyelination [25,35].

To study gut microbiota interactions with the host response, the germ-free (GF) animal model is an important tool [34]. Researchers observed that laboratory mice that develop in a germ-free environment have incomplete development of the CNS [36]. It is now known that administering certain bacteria allows these laboratory mice to have a normal development. These discoveries have opened up new horizons for research on how the gut and the brain communicate.

Recent studies have shown that the intestinal environment is directly involved in MS pathogenesis [26,37,38]. In the last decade, studies using in vivo models or human subjects have proved that different bacteria and fungi are present in the CNS in MS patients, e.g., an infection with opportunistic bacteria can induce changes in the inflammatory status of the body that promote an unbalanced proinflammatory response. Bacteria-related neuroinflammation can lead to demyelination and neurodegeneration [39,40].

Germ-free animal models produced clear evidence of the microbiota’s impact, both in the developmental and later post-developmental stages of the CNS. However, these influences seem to have both favorable and unfavorable facets. These findings strengthen the hypothesis that the intestinal environment is a critical factor in MS pathogenesis.

On the other hand, the gut microbiota from MS patients have been shown to accelerate autoimmunity via Th17 cells in a transgenic mice model of spontaneous autoimmune encephalomyelitis (EAE) [27,41]. Other studies established that the gut microbiota play an important role in the neuroinflammatory response by controlling T cell immunity through balancing Treg/Th17 [42]. However, studies report that gut microbiota signals activate autoreactive T cells in response to a specific myeline oligodendroglia glycoprotein (MOG) or MOG-like peptides present in some gut microorganisms (e.g., *Lactobacillus reuteri*) [43].

### 1.4. Are the CNS Cells Impacted by Metabolites Derived from the Gut Microbiota?

Recent studies report that small molecules released by gut bacteria, called bacterial metabolites, have altered levels in MS [44,45,46]. These metabolite profiles can be valuable biomarkers in MS. Many molecules are described as having altered plasma concentrations. All of these molecules that are involved in immune response regulation have gained increased attention. Short-chain fatty acids (SCFAs) are directly impacted by dietary components and gut microbiota and have immunoregulatory properties by controlling regulatory T cell (Treg) migration to pathological sites [46,47]. Levels of SCFAs, such as acetate, propionate, and butyric acid, are altered in MS. All these metabolites are produced by gut bacteria through different pathways [46]. Some studies report that SCFAs have a neuroprotective effect on CNS cells by activating neurogenesis and through anti-inflammatory and antioxidative actions [48]. The opposite effect was reported by others, who found that SCFAs can pass the BBB and are involved in immune activation and neuroinflammation in the brain [49,50]. On the other hand, propionate and butyrate was reported to stimulate antibody production by modulating B cell differentiation [51].

Alteration in the levels of other metabolites, such as bile acid metabolites, that results from cholesterol metabolism in the astrocytes and microglia, is associated with neuroinflammation in EAE animal models [52]. The appropriate level of bile acids can prevent the microglial-astrocytic switch from a protective to a neurotoxic phenotype in MS [53].

In addition, polyamines are a class of active metabolites produced by gut bacteria that control the innate immune response in the CNS and many important functions (e.g., cell differentiation and growth, modulation of ion channels, and autophagy) [54]. An alteration of the polyamine levels can be detrimental for CNS cell functions through chronic activation of astrocytes, alteration of calcium ion influx, and increased catalase activity [55].

### 1.5. Brief Overview on Immune Response in MS

It is now well understood that immune cells can activate the cellular and molecular pathways underlying neurodegeneration. The MS pathophysiology is characterized by neuroinflammation, demyelination, and glial scar formation [56,57]. Here, we have four disease progression stages in MS: clinically isolated syndrome, relapse–remitting stage (RRMS), primary progressive stage (PPMS), and secondary progressive stage (SPMS). In every stage of MS disease, an active demyelination process followed by neurodegeneration is accompanied by neuroinflammation [57]. Inflammation is mediated by T and B immune cells, activated microglia, and macrophages.

In early stages of MS, clinically isolated syndrome and RRMS, there is an overactivation of the inflammatory response that leads to demyelination. In the PPMS and SPMS, the basic processes remains the same but become more expansive [57]. At this point, the pathophysiological profile is dominated by irreversible neurodegenerative lesions. Many cellular mechanisms that underlie neurodegenerative lesions can be triggered by activated inflammatory cells and their active molecules (e.g., pyroptosis, demyelination, autophagy, and astrocyte disfunction) [58].

It is well accepted that MS lesions are characterized by peripheral autoreactive immune cells that infiltrate the CNS through BBB disruption [59,60]. These autoreactive immune cells include regulatory T cells (e.g., Th1, Th17, CD8+ T cells), activated B cells, and activated plasma cells that release autoantibodies [61]. However, the overactivation of exogenous and endogenous CNS immune cells leads to neuroinflammation and neurodegeneration [15]. Autoreactive CD4+ T cell invasion of the CNS is the first event that underlies MS disease. Th17 cells express the C-C motif chemokine receptor 6 (CCR6) that binds to its ligand (CCL20) expressed by BBB endothelial cells and pass to the CNS [62]. Activated CD4+ Th17 cells release active molecules such as IL17 and granulocyte–macrophage colony-stimulating factor (GM-CSF) and trigger microglia and macrophage activation. Mononuclear phagocytes activated by GM-CSF can migrate into the CNS. GM-CSF produced by activated cells can induce ROS production by activated microglia that mediate neuronal damage [62]

In addition, CD4+ Th1 cells pass the BBB into the CNS and release gamma-interpheron (IFN-γ), which potentiates the activated microglia to release IL12 and initiate a CD8+ cytotoxic T cell response [61]. In MS, IFN-γ can induce major histocompatibility complex class I molecules (MHC I), an important regulator of immune response related to pathogen defense [63]. Increased activity of Th1 and Th17 cells releases other pro-inflammatory molecules (e.g., cytokines, interferons, and tumor necrosis factor) leading to CNS damage [64]. In addition, Th17 cells and CD8+ cells are responsible for IL17 production. IL17 is a powerful pro-inflammatory cytokine that contributes to neutrophil and monocyte recruitment at the CNS level [64]. Furthermore, IL17 was reported to increase oxidative stress at the level of vascular endothelial cells through its receptor (IL17R) [65]. Interleukin 6 (IL6) and IL22 are other important pro-inflammatory cytokines that regulate the balance between Th17 cells and Treg cells [64]. Th1 and Th17 cells create a pro-inflammatory environment that attracts peripheral monocytes and promotes neuronal damage.

In a normal immune response, B cells fight alongside T cells against pathogens. B cells are involved in adaptive immune responses to microbes that preserve healthy gut microbiota. They act through immunoglobulin A (IgA) production. Alteration of these local defense mechanisms leads to gut dysbiosis and increases the risk of autoimmune diseases in genetically predisposed individuals [29]. In addition, gut dysbiosis alters peripheric tolerance for the B cells and favors tissue damage far away from the gut. In MS, after passing the peripheric tolerance, B cells are transformed into a CXCR3+ population able to infiltrate the brain [66]. B cells release antibodies against myelin sheets that damage them through a complement-mediated pathway [58]. However, recent findings suggest that B cells contribute to MS diseases through autoantibody-independent mechanisms. Central and peripheral tolerance mechanisms control the autoreactive B cell development [67]. A defective peripheral tolerance is reported in MS patients [68].

The activated microglia phenotype is associated with active demyelination in MS [69]. Activated microglia release pro-inflammatory mediators (e.g., ROS, NO, and peroxynitrite) with an important role in the phagocytosis of mielin, antigen T cell presentation, and cytokine production, and at the injury site [57].

However, secondary mechanisms that lead to neurodegeneration are related to pro-inflammatory mediators (interleukins, NO, or ROS). Tumor necrosis factor α (TNF α), a pro-inflammatory cytokine, can display a protective or detrimental role for neuronal cells, according with other molecule levels (e.g., increased NO level) or NFkB pathway activation [70]. NO produced by activated microglia alter energy production at a cellular level, which leads to increased intracellular Ca^2+^ and damage to neuronal cells. In addition, TNF α promotes the apoptosis or pyroptosis of neuronal cells [58].

Acute demyelinated lesions in MS progress into chronic active lesions with a astroglial scar core and inflammatory lesions [71]. Astrocyte cells are necessary for normal function of CNS cells, but a neurotoxic phenotype can develop that is responsible for MS progression. Within the immune system, CNS cells such as microglia and astrocytes can play an important role in MS development and progression [72]. Astrocytes maintain BBB function and neurotransmitter levels. One important role is to regulate the glutamate level by controlling key enzymes involved in glutamate production. Astrocytes express glutamate transporters and this expression is altered in MS patients. An increased extracellular level of glutamate leads to glutamate-mediated excitotoxicity and neuronal death [58].

## 2. Oxidative Stress: The Impact of Gut Microbiota on Mitochondrial Function and Neuroinflammation

Oxidative stress is not only dependent on the concentration of ROS, reactive nitrogen species (RNS), or reactive sulfur species (RSS), but it also depends on the duration of oxidative stress exposure [73]. It is sensible to assume that there is a time window for proper interventions in MS management.

ROS are produced by activated microglia and macrophages and contribute to mitochondrial disfunction in MS. Mitochondrial disfunction triggers neuronal cell death. Mitochondrial lesions related with an increased oxidative stress may underlie the important MS progression features such as oligodendrocytes pyroptosis, neuronal degeneration, demyelination, and lack of restorative processes [57]. The oxidative stress is driven by increased microglial ROS production and microglial inflammation in MS diseases [57]. However, gut dysbiosis is associated with an increased level of bacterial lipopolysaccharides (LPSs) and its proteins, such as LBP-LPS binding protein, at the CNS level. LPSs act as exotoxin and trigger B cell activation and cytokine production. Recent data report that LPSs act as caspase activators and inflammasome activators that lead to pyroptosis [74].

The entire scenario is made more complex due to the intricate interrelationship between gut dysbiosis, oxidative distress, and neuroinflammation. Many neurodegenerative and autoimmune diseases, including MS, are caused by at least one of these interactions.

### Is the Gut Environment Involved in CNS Mitochondrial Dysfunction?

The mitochondria play an essential role in gut–brain axis communication. Some dietary components and gut microbiota metabolites interact with the mitochondrial metabolism [75,76]. This leads to mitochondrial dysfunction, energy failure, and increased ROS production, which are maintained in a vicious cycle. Increased ROS production leads to systemic inflammation through inflammasome activation [77]. Since mitochondria have their own DNA (mtDNA), they are very sensitive to oxidation and genetic and epigenetic modification. Such mtDNA alterations were reported in MS [78].

## 3. Redox Signaling Pathways That Target GIB/BBB Integrity and Inflammasome Activation in MS

**Figure 1:**
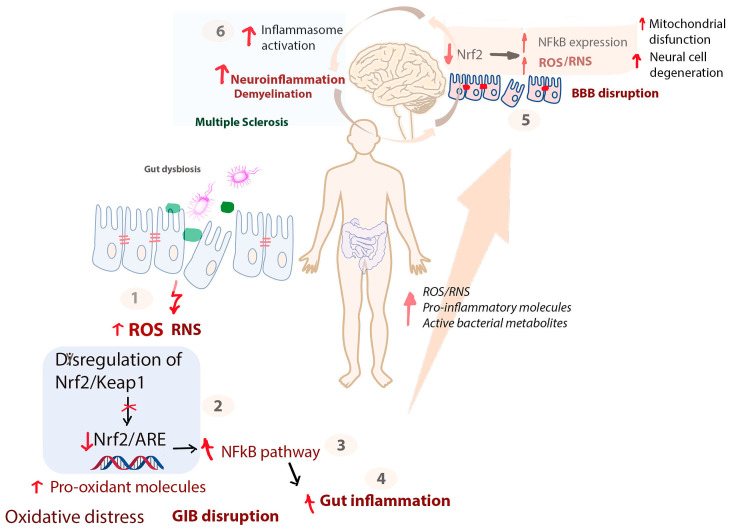
Redox signaling pathways involved in GIB/BBB integrity and inflammasome activation in MS. Gut dysbiosis, irrespective of its cause, leads to: (1) increased ROS/RNS concentration; (2) dysregulation of the Nrf2/Keap1 protective pathway; (3) decreased expression of Nrf2/ARE transcription factors, which leads to activation of the NFkB pathway, in turn promoting gut inflammation and leading to GIB disruption; (4) GIB disruption increases the permeability to small active compounds released by gut bacteria and makes it easier for bacteria to invade by lowering the local immune defense; (5) active gut microbiota metabolites trigger the BBB disruption and increase ROS/RNS and pro-inflammatory molecule levels through inhibition of Nrf2; and (6) increased ROS levels in the brain induce mitochondrial disfunction and inflammasome activation.

The gastrointestinal tract is populated with a trillion commensal bacteria that maintain gut barrier integrity and pathogenic bacteria [79]. Both types of bacteria modulate their mitochondrial activity and affect ROS production directly or indirectly through active metabolites (e.g., SCFA, formyl-peptides) [80]. Gut dysbiosis is an important contributor to MS due to excessive ROS production by bacterial metabolism or by activation of NADPH oxidase. Increased ROS alters intracellular signaling pathways and promotes inflammation in MS patients [81,82]. ROS are important regulators of innate immune response through inflammasome activation [82,83]. ROS stimulate the release of proinflammatory cytokines that promote inflammation and inflammatory-mediated apoptosis (pyroptosis) (Figure 1). T and B cells are activated by ROS commensal bacteria display antioxidant and anti-inflammatory properties. In contrast, pathogenic bacteria promote oxidative stress [84]. Depending on their concentration, ROS act through non-enzymatic-mediated oxidative damage or as redox signaling pathway modulators of some important pathways, such as the nuclear factor erythroid 2-related factor (Nrf2) pathway, TLR pathway, or NF-kB pathway, in the intestinal epithelial cells. Some of these pathways are involved not only in redox signaling, but also in inflammation. A proper interaction between gut bacteria and immune system cells has a protective role by controlling ROS production.

Nrf2 is a key transcription factor involved in the regulation of cellular antioxidative defense and in the cellular response to various stressors. Nrf2 decreases ROS concentration, which is associated with the inflammatory response and diminishes neuroinflammation. In addition, Nrf2 transcription factors are key regulators of the mitochondrial function, acting through peroxisome proliferator-activated receptor gamma coactivator 1 alpha (PGC-1alpha) [85]. There is a specific relationship between the gut microbiome, mitochondrial dysfunction, and the host immune response in MS. Some bacteria, such as *Clostridium* spp. or *Lactobacillus rhamnossus*, can inhibit NFkB-mediated inflammation through their metabolites, which act as immune response controllers [86,87,88]. Animal model studies showed that activation of NFkB within physiological limits plays a protective role in the gut [89]. This protective role is linked to the immune defense against pathogens [90]. There are significant adverse effects if NFkB is abolished [91]. Whether we can safely interfere with this signaling pathway remains to be determined.

In the neural tissue, Nrf2 is expressed in a larger amount in microglial cells that are involved in the inflammatory response to lesions or infection. When inflammation appears, microglia are activated and can participate in neurodegeneration in MS. When macroglia produce Nrf2, an anti-inflammatory and anti-immune response is activated and can trigger MS development [92]. Given this fact, Nrf2 displays a bidirectional effect in the inflammatory process, acting as a messenger and mediator that promotes neuroprotection [93,94]. Oxidative distress decreases Nrf2 expression and promotes oxidative stress by decreasing NADPH quinone dehydrogenase 1 (NQO1) protein levels [95]. NQO1 enzymes protect the cells from peroxidation through the modulation of the quinone metabolism (e.g., vitamin K). In the last decade, many research studies proved that Nrf2-ARE pathway activation by a chemically active compound or cellular stress maintains BBB integrity and function in neurodegenerative diseases [94,96,97,98,99]. Decreased Nrf2 expression can lead to chronic oxidative distress, which increases the permeability of the BBB, and therefore exposes the brain to various bacteria and active metabolites [100]. Decreased Nrf2 transcription factor levels lead to increased NFkB expression and promote neuroinflammation through canonical or non-canonical NFkB pathways, while also having an impact on autophagy and MS development [101].

Oxidative stress releases the Nrf2 transcription factor from its combination with KEAP1 through a mechanism that is not fully understood. In MS, increased Nrf2 levels inhibit the NLRP3 inflammasome activation by acting on the thioredoxin-interacting protein (TXNIP) [102]. Nrf2 promotes the dissociation of TXNIP from thioredoxin 1 (Trx1) in response to an increased level of pro-oxidant molecules and diminishes inflammation through control of the cellular redox state [102]. In contrast, Kobayashi and colleagues reported that Nrf2 increases inflammation through the induction of proinflammatory cytokines mediated by lipopolysaccharide-related transcriptional activation of gene expression [103].

### 3.1. Is MS an Inflammasome-Related Autoimmune Disease?

Some studies on humans have the objective of answering this question with a genomic approach. Genomic studies have identified an associated variant in the *NLRP3* gene in RRMS patients [104]. Additionally, genetic variants in inflammasome transcription factors, *NLRP1*, caspase 1, and alteration of pro-apoptotic gene expression were seen in sporadic and familial cases of MS [105,106]. However, the role of inflammasomes in MS is not fully understood.

The NLRP3 inflammasome is controlled by mitochondrial reactive oxygen species (ROS) and oxidized mtDNA, and ketone bodies block it. Ketogenic and fasting diets improve MS outcomes through NLRP3. These interventions are proven to be effective in animal models and in humans with neurodegenerative diseases, including MS [107,108].

Recent studies have shown that the Nrf2/KEAP1 signaling pathway has been implicated as a key factor in MS-associated depression. Since many neuropsychiatric conditions are associated with increased inflammation and oxidative stress, overactivation of Nrf2 is a critical adaptive mechanism aimed at controlling this process [109]. The Nrf2/KEAP1 pathway is an important tool to control redox homeostasis and improve functional outcomes in depression associated with MS [110]. Increasing evidence shows that Nrf2 activation is responsible for increased brain-derived neurotrophic factor (BDNF) levels, which lead to neuroprotection [111]. In addition, Nrf2 is involved in ferroptosis and autophagy and can be involved in cytoprotection and mitochondrial integrity [112]. This pathway seems to play an important role in controlling stress resilience, which can improve clinical symptoms [113]. Gut microbiota can play an important role in this pathway, which has been reported to be associated with stress, anxiety, and learning difficulty [114].

Inflammasomes may be a promising target for oxidative-stress-related neuroinflammatory control. Currently, the most studied inflammasome in MS is NLRP3. NLRP3 can be activated by a large variety of molecules. One such molecule is nigericin, released by Gram-positive bacteria. Nigericin can activate the NLRP3 inflammasome and induce oxidative-stress-related inflammation [115]. Other bacterial molecules involved in the NLRP3 pathway activation have not been extensively studied.

Viral particles or bacterial or environmental toxins can induce an alternative activation pathway of NLRP3 via increased ROS production or mtDNA oxidation. All of these events allow inflammasome activation to promote gasdermin D (GSMD) activation and pyroptosis [116]. GSMD is a key protein that controls cytokine production and cell death. GSMD has pore-forming activity at the cell membrane level, which leads to interleukin 1 cytokine secretion in the extracellular space and promote pyroptosis [117].

While other inflammasome pathways are targeted by bacteria through PAMPs, NLRC4 is activated by different bacterial components (e.g., flagellins, inner rods, or needles) that are released by Gram-negative bacteria such as *Salmonella* spp. This activation of NLRC4 leads to inflammatory death. NLRC4 plays an important role in the protection of mucosal bacteria at the gut, stomach, or lung level [118,119].

Some other inflammasomes, e.g., absent in the melanoma 2/AIM2 pathway, have also not been studied in MS. However, several studies report the AIM2 pathway is activated by ROS production independent of NLRP3, most likely by bacterial cell antigens and bacterial DNA [120,121,122].

Many studies have reported divergent results, potentially due to the small number of patients and large inter-individual variability. So far, many studies have not investigated inflammasomes, despite looking at proinflammatory status in MS [60,123].

### 3.2. Is the Exposome the Missing Piece in a Comprehensive Approach to MS?

It is known that during fetal development, our brain is exposed to an increase in inflammation that favors new placental blood vessel formation in order to sustain the increased demand for oxygen [124]. The physiological disturbance of the pro-oxidant/antioxidant balance is essential during the developmental period [125] as it influences gene expression and activates important developmental pathways [126,127]. Some exposomes appear during the developmental period and can be triggered later in life by environmental exposures (e.g., smoking, food composition, or socio-economic factors) [128,129].

Genetic tools can provide valuable information about susceptibility to diseases, including MS. MS-specific gene variants have been described, but only a few people who carry these potential pathogenic gene variants develop the disease. Despite recent advances in genetics, the MS risk factors cannot be fully explained by genetics. In this light, in 2005 a complex interaction between life-course environmental factors that modulate biological responses was defined as an exposome [130,131,132]. Recently, the complete exposome concept has included the link between internal exposomes and external exposomes [131]. Internal exposomes are related to the homeostasis of the human body and include cell metabolism, gut microbiome, inflammation, and the redox system [131]. Since external exposure is related to specific environmental factors (e.g., environmental pollution, lifestyle factors), it belong to external exposomes [131]. However, internal and external exposomes are partially overlapped and strongly interrelated. The exposome concept includes all these interactions [131]. The exposome scenario becomes more complex in neurological diseases due to very dynamic CNS changes in response to environmental factors [133]. This multimodal approach is essential to understanding the complex relationship between the exposome and its biological consequences in MS [131]. Recent studies report that changes in the environment can improve or aggravate functional outcomes in CNS diseases [132,134].

MS is known to be influenced by environmental and lifestyle factors. In this light, the MS exposome is highly relevant [135]. Redox signaling is also influenced by environmental factors, but how these interactions modulate gut microbiota and the host response consequences remain elusive. Active exogenous molecules may be contained in food, ingested willingly or accidentally, as food composition has suffered dramatic changes due to environmental pollution. In addition to cigarette smoking or heavy metal exposure as the main environmental exposomes, in the last decade, it has been reported that there is a strong association between pesticides and an increase in ROS production and an inflammatory response in many diseases, including MS [136,137,138].

Pesticide exposure can lead to redox imbalance due to a chronic increase in ROS/RNS production [139,140,141,142]. In in vivo studies, it has been reported that pesticides increase ROS/RNS via an increase in nitric oxide production by activating iNOS transcription [143,144]. Depending on the class of pesticides, these compounds can act on different signaling pathways [145]. Some pesticides can act as modulators of the Keap1/Nrf2/ARE pathways and the NFkB pathway [138,146,147]. Chronic exposure to some organophosphorus pesticides can lead to the overactivation of microglial cells and an increase in ROS production through NADPH oxidase (NOX) activation, which is associated with an increase in proinflammatory cytokine production [148]. However, these studies on pesticide mechanisms are subject to bias due to the diverse chemical and structural properties and the heterogeneity of different pesticide mixtures. Human studies have been limited to a small cohort, and even fewer studies have been related to MS or sex-related MS differences linked with the exposome. Further standardized studies need to be performed on MS in order to establish the molecular mechanism that underlies the disease’s onset.

**Figure 2:**
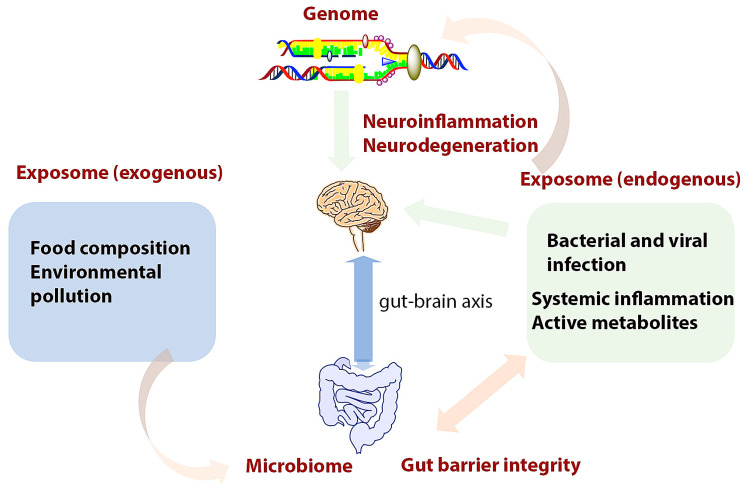
Complex interrelationships involved in GIB/BBB integrity, inflammation, and neurodegeneration that need to be included in MS management and therapy. Exogenous exposomes interact with gut microbiota and modulate the gut microbiome; this interaction can be either protective or damaging to microbiome composition and GIB. The intestinal disbalance triggers neuroinflammation and neurodegeneration via the endogenous exposome and the gut–brain axis. These biological processes are conditioned by host genome.

On the other hand, environmental pollutants induce gut microbiota changes that can trigger the GIB and, subsequently, BBB disruption (Figure 2). However, a large variety of environmental chemical compounds (e.g., xenobiotics and food additives) are metabolized by gut microbiota through specific enzymatic systems. Some studies have reported that there is an alteration of these mechanisms that originate in the perinatal period [149].

It is well established that exposome profiles undergo permanent changes over time, depending on environment or lifestyle [150]. Exposome-wide analysis studies performed during the lifespan, or at least extended periods of time, may capture an accurate individual exposome risk profile for MS. The exposomes of individuals in the same geographic area can be differently influenced by the same exogenous exposome [150]. The link between these and MS was intensively studied during the last decade, and it is not the focus of this review. External exposomes are not limited to the lifestyle or geographic area, and it is crucial to have a deeper understanding of how other external exposomes can influence the endogenous ones. Regional approaches may further capture specific air and water pollution in different geographic areas.

Exposomes can impact the response to disease-modifying therapy and, therefore, the progression of MS. Therefore, there is interest in at least capturing it, if not, preferably, modulating it. Recent advances in technology have allowed some progress in exposome measurement, contributing to MS epidemiology. Furthermore, exposomes such as lifestyle (e.g., stress, physical exercise, nutrition) can be easily self-monitored using widely available technology such as mobile phones. Recent mobile applications were developed for MS patients to track fatigue or cognitive impairments [151,152].

A better understanding of the interplay between the individual exposomes and the host genome is needed. Multi-omics approaches have been described in a recent study (Human Early Life Exposome Project). The authors analyzed the molecular profile subsequent to exposure to different exposomes during childhood [153]. This study reported changes that affected all the omics levels (genome, transcriptome, and epigenome), but significant changes were reported at the serum metabolite level. Cellular responses to exogenous and endogenous exposomes are linked with host genetics and disease phenotypes. This opens new perspectives for uncovering molecular signatures of diseases related to exposomes. The method is also enticing for its potential to identify biomarkers with clinical impact, in the diagnosis or prediction of disease progression.

A better understanding of these complex interactions will allow us to establish which factors underlie cellular and molecular mechanisms in MS. In addition, we can understand how these factors can be better controlled through personalized therapy. Investigating the exposome has the potential to change public health policies and ultimately meaningfully impact disease outcomes. This is essential for clinical practice, acknowledging that earlier treatment is associated with a better outcome in MS [154].

## 4. Modulation of Redox Signaling and Inflammation by Gut Microbiota Active Molecules

Exposomes such as chemical compounds or microorganisms have a significant impact on autoimmune diseases [155], including MS. Gut exposomes are represented by active compounds produced directly by the gut microbiota or produced through the biotransformation of compounds such as dietary constituents and xenobiotics [156,157]. Various gut microbiota active compounds belonging to different chemical classes have been studied in the last decade in relation to MS.

Tryptamine is a metabolic by-product of tryptophan that commensal bacteria releases at the intestinal level. It belongs to the indolamine organic compound family that decreases inflammation by inducing microbiota changes and increasing the butyrate level [158]. Tryptamine acts as a neuromodulator and has antioxidant properties at the cellular level. A natural derivative of tryptamine, 5-hydroxy-tryptamine (5-HT), is essential for CNS regulation and improves mood in humans. In MS patients, 5-HT deficits leading to depression are frequently reported. Currently, serotoninergic modulators such as SSRIs are a viable treatment option [159]. These modulators have additional effects on microglial activation and decrease oxidative stress and neuroinflammation [160].

Interestingly, another study reports a second tryptamine-derived organic compound to be effective in MS therapy. N-acetyl-5-methoxytrypatmine (melatonin), a second tryptamine derivative organic compound, is reported to be effective in MS. Melatonin acts as a signaling molecule at the CNS level and regulates immune response, oxidative stress, and apoptosis [56,161]. A recent review describes that melatonin interactions with the gut microbiota lead to oxidative stress and inflammation [56]. Similar to tryptamine, melatonin deficiency is reported to be involved in MS comorbidities (e.g., fatigue or depression). It increases the antioxidant capacity of neuronal cells by increasing the CAT, SOD, GPx, and GSH levels [56]. The in vitro study of Cheng and colleagues showed that microbial-derived antioxidant molecules reduce oxidative-stress-related inflammation through Nrf2 as a modulator of the ROS/NLRP3/IL1beta signaling pathway [162]. Since serotonin and melatonin levels are strongly influenced by light exposure, we question if there is a link between external exposomes (e.g., geographic area, stress, and/or xenobiotics), the gut microbiome, and these active compounds’ deficits in MS.

Indoles are also produced by the gut microbiota, from tryptophan. Its metabolism results in a series of compounds (e.g., indole-3-propionic acid, indole acetic acid) that cross the BBB and act as neuroprotectors by decreasing oxidative stress [163].

Research studies have shown that gut dysbiosis is associated with a lack of other important organic compounds, such as amino acids. The lack of the amino acid cysteine due to intestinal bacteria can contribute to a decrease in glutathione synthesis and increased oxidative stress [164].

Other metabolites are short-chain fatty acids (SCFAs). Acetate, butyrate, and propionate produced by the gut microbiota are important factors that influence the immune host response, demyelination/remyelination balance, and oxidative distress [30]. Research studies have reported the opposite effect of short SCFAs compared with that of long-chain fatty acids (LCFAs). Dietary LCFAs promote a proinflammatory status through Th17 cell activation. In contrast, SCFAs released from the gut microbiota metabolism of dietary fibers from food act as anti-inflammatory players through anti-inflammatory cytokine production and restore BBB integrity in the GF animal model [165,166,167]. There is a very fine balance between pro- and anti-inflammatory regulators, and the gut microbiota are a key factor in this equation [168,169]. SCFAs, similar to melatonin, can modulate redox signaling through the Nrf2 pathway and are involved in the host response linked to the gut microbiota [167].

Active compounds can be measured in biological samples using metabolomics. A recent review of literature data focused on the metabolomic profiles of MS [170]. It is important to establish the compound’s source of origin as either endogenous or produced by the intestinal microbiota [171]. The measurement of gut microbiota active compounds is challenging due to their similarities with endogenous ones. A recent metabolomics study performed by Neveu and colleagues created, for the first time, a gut-microbiota-specific exposome database [171]. By interconnecting all available databases, it will be possible to establish interactions between exposomes, genes, and the metabolic response of the host in MS. This signature can better predict the disease’s outcome and identify new protective active compounds.

Active compounds with antioxidant and anti-inflammatory properties are currently used to prevent or improve MS outcomes. They need to be metabolized by the gut microbiota. For example, some flavonoids are metabolized by the gut microbiota and microbiota-derived active compounds are released. Rutin is metabolized in the gut by several gut bacteria (*Lactobacillus* spp., *Bifidobacterium* spp., *Enterobacteriaceae*, and *Lahnospiraceae*) to quercetin and its derivatives [172,173]. Gut dysbiosis affects these bacteria, thus leading to rutin absorption deficiencies. Quercetin is a chemical compound that displays anti-inflammatory, antioxidative, and neuroprotective properties in many autoimmune diseases, including MS. It acts on the NFkB pathway, increasing the GSH level and decreasing microglial activation [174,175,176,177]. Gut microbiota enhances quercetin absorption by deglycosylation to form aglycone (*Lactobacillus* spp., *Bifidobacterium* spp., *Clostridium perfringens*, *Streptococcus, Bacteroides*). Similar to quercetin, kaempferol is converted by *Lactobacillus paracasei* into small active compounds that can be easily absorbed [178,179,180]. In addition, certain types of anthocyanins and flavones need to be transformed by the gut microbiota in order to be absorbed [181].

Vitamin D protects the neural cells from ROS production by increasing their antioxidant capacity [182]. Recent findings report that *Bacteroides* spp., *Bifidobacterium* spp., and *Akkermansia* are increased by vitamin D [183,184]. In addition, gut microbiota composition interacts with vitamin D response and promotes “resistance” [185]. Similar to vitamin D, ascorbic acids or vitamin E increase *Firmicutes*, and decrease *Actinobacteria* and *Bacteroidetes* [163]. In addition, vitamin D and E modulate the metabolic activity of the gut microbiome.

In the multimodal approach to MS, a mixture of gut microbiota active compounds rather than a single antioxidant can be used with clinical benefits. However, here, we have to take into account which combination is necessary to restore the balance.

## 5. Clinical Trials That Target Gut Microbiota in MS Patients

Oxidative distress that can be potentiated by dietary factors that are closely related to gut dysbiosis in MS patients. Many of these studies report foods that are rich in antioxidant and anti-inflammatory compounds as important disease-modifier interventions [186,187,188,189].

It is still challenging to distinguish between nutraceuticals, metabolic changes due to a decrease in unhealthy dietary components, and metabolic changes. It can be due to active metabolic compounds released by the gut microbiota. It is even more difficult to identify which of the “good bacteria” are key players in this mechanism in humans. However, there is a vicious cycle between nutraceuticals, the type of bacteria that are favored by diets, and the metabolic active compounds released.

In the last decade, many studies have demonstrated successful strategies or interventions using in vitro and in vivo MS models. Some of these strategies include probiotic or symbiotic supplements and fecal microbiota transplantation. These interventions were tested in clinical trials (Table 1).

Probiotics and prebiotics act on the inflammatory stress response [195]. Unanswered questions are related to the appropriate timing for administration initiation, therapy length, and appropriate dosages. Interventional therapies must be designed according to age, exposure, and particular host responses (individual microbiome, genome, transcriptome, and metabolome). Any chosen active compound used in this context should, first, restore the gut dysbiosis and, second, stimulate the favorable bacteria. It is essential to measure a personal gut microbiota profile. Last but not least, monitoring exposure and lifestyle is a must in a multimodal approach. Personalized therapy design for MS according to environmental exposure during lifespan, microbiome profile, genetic and epigenetic risk factors, and MS disease stage significantly increases the chance of achieving clinical benefits.

## 6. Conclusions

A growing amount of evidence shows that targeting the gut microbiota can be a promising strategy for the management of MS disease. Overall, we believe that the new and exciting discoveries summarized above suggest that gut microbiota have the potential to influence the host intestinal environment and communicate with the brain. Some key points are highlighted here. First, the gut microbiota can over-activate the innate and adaptive immune responses and contribute to MS pathophysiology. This interaction between microbiota and host immune response occurs via the gut–brain axis.

Second, gut dysbiosis mediates the oxidative stress and is associated with inflammasome activation. Activation of the inflammasome is triggered by bacterial active metabolic compounds and promotes inflammatory-related cellular death called pyroptosis, which leads to neurodegeneration. Here, we presented how ROS production and the inflammasome pathway can link the gut microbiome and the brain. This inflammasome-mediated communication between the gut and the brain is ROS-dependent and can be targeted for new therapy in MS patients. Third, since MS pathophysiology cannot be described only by genetics, it is generally recognized that there is a complex interaction between genetic and environmental factors. The exposome concept has gained increased attention. We described the role of relevant MS-related exposomes that involve the gut–brain axis. We conclude that future studies related to exposomes are required in order to better understand the development of MS. Modulation of the gut–brain axis by controlling gut microbiota composition can decrease oxidative-stress-related inflammation and attenuate neurodegeneration in MS patients. However, to assess the importance of the microbiome as a disease biomarker and the effectiveness of microbial interventions, translational approaches and relevant clinical trials are necessary. These are required to better understand the temporal and causal relationships between gut microbiota and the development of MS. Overall, we conclude that a multimodal approach to MS management will help to better understand the onset of MS and improve its outcome.

## Figures and Tables

**Table 1:** Human trials that target gut barrier integrity in MS.

Clinical Trials	Subjects	Intervention or Exposure	Dosage/Time	Outcome
IRCT20141108019853N7 randomized clinical trial [190]	*n* = 70; 35 MS patients (RRMS, PPMS, PRMS); 35 placebos	Cointervention with: - *Symbiotic* supplements with 8 strains: *Lactobacillus* spp. (*L. plantarum*, *L. bulgaricus*, *L. casei*, *L. acidophilus*), *Bifidobacterium* spp. (*B. infantis*, *B. longum*, *B. breve*) and *Streptococcus thermophilus*; - *Anti-inflammatory and antioxidant rich diet* according to the dietary inflammatory index and oxygen radical absorbance capacity.	4.5 × 10^11^/day with fructooligosaccharide (100 mg)/4 months	Primary outcome: fecal calprotectin level decreased compared with that of placebo.
IRCT20161022030424N1 randomized clinical trial [191]	*n* = 50; 25 MS patients; 25 placebos	*Probiotic* supplements: *Saccharomyces bullaria.*	10^10^ CFU/day with magnesium and lactose stearate (100 mg)/4 months	Primary outcome: - Inflammatory markers: serum C-Reactive Protein (CRP); - Oxidative stress markers: malondialdehyde (MDA), total antioxidant capacity (TAC); - Quality-of-life-related markers: mental health, pain, fatigue.
IRCT20181210041918N1 randomized clinical trial [192]	*n* = 60; 30 RRMS patients; 30 placebos	*Probiotic* supplement with 14 strains: *Bacillus subtilis*, *Bifidobacterium* spp. (*B. bifidum*, *B. breve*, *B. infantis*, *B. longum*), *Lactobacillus* spp. (*L. acidophilus*, *L. bulgaricus*, *L. delbrueckii*, *L. casei*, *L. plantarum*, *L. rhamnosus*, *L. salivarius*, *L. helveticus*), *Lactococcus lactis* and *Streptococcus thermophilus*.	4 × 10^9^ CFU/day for 12 weeks	Primary outcome: - Inflammatory markers: C-reactive protein (CRP), tumor necrosis factor alpha (TNFα), gamma interferon (IFNγ), interleukins (IL17, IL35); - Other markers: tumor growth factor beta (TGFβ), forkhead fox P3 (FOXP3); - Diet and physical exercise.
IRCT2017082234497N2 randomized clinical trial [193]	*n* = 48; 24 RRMS patients; 24 placebos	*Probiotic* supplement with 4 strains: *Bifidobacterium* spp. (*B. infantis* and *B. lactis*), *Lactobacillus* spp. (plantarum and fermentum).	2 × 10^9^ CFU/day for 16 weeks	Primary outcome: - Oxidative stress biomarkers: MDA, 8-hydroxy-deoxyguanosine, SOD, GSH, TAC; - Inflammation biomarkers: hs-CRP, IL10, IL6, TNFα and NO; - Other measurements: EDSS, insulin level, fasting blood glucose, general health, depression.
NCT03183869 pilot randomized clinical trial [194]	*n* = 9 RRMS patients randomly divided into 2 subgroups: subgroup 1—early intervention (n = 4); subgroup 2—late intervention (*n* = 5); *n* = 2 healthy donors	*Fecal microbiota transplantation* from healthy donors: - In subgroup 1 for up to 6 months (1 intervention/month) and follow up for the other 6 months; - Subgroup 2 followed a reversed protocol (first 6 months for monitoring and fecal transplant after this period for the other 6 months).	NA/1 intervention per month for 6 months	Primary outcome: panel of 25 pro- and anti-inflammatory cytokines; safe and tolerable.
NCT03594487 non-randomized clinical trial	*n* = 30; RRMS patients and control	*Fecal microbiota FMP30 transplantation* after 5 days of preconditioning with antibiotic treatment.	NA/12 weeks	Primary outcome: fecal microbiota changes and incidence of adverse effects; secondary outcome: serum immunoglobulin levels and CD19+ and CD20+ B cells.

## Data Availability

Not applicable.
